# Supplementary material for: A Non-Invasive Deep Photoablation Technique to Inhibit DCIS Progression and Induce Antitumor Immunity
Source: Cancers (Basel). 2022 Nov 23;14(23):5762. doi: 10.3390/cancers14235762 (PMC9735847; doi:10.3390/cancers14235762)
Supplement: Supplementary file 1 [file cancers-14-05762-s001.zip › cancers-2031606-supplementary.pdf]

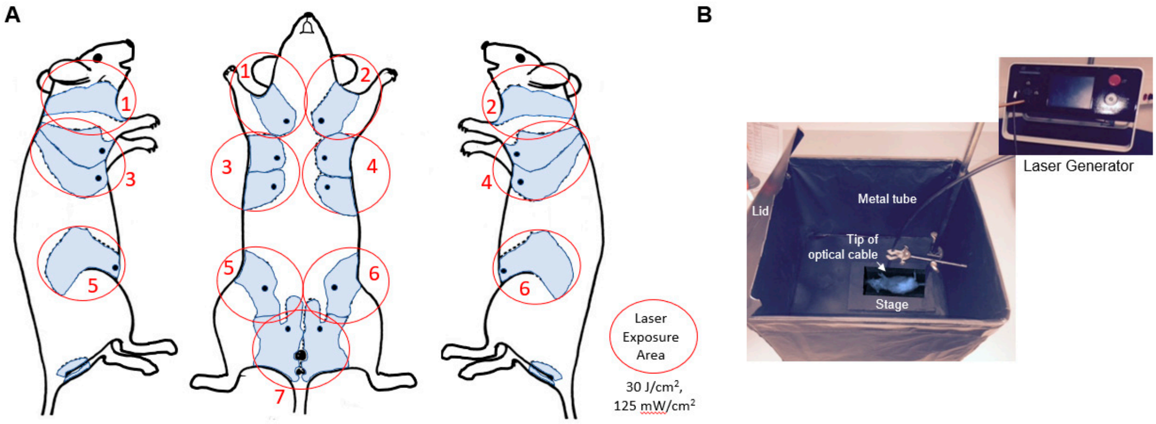

Supplementary Figure S1. Laser Exposure Areas for the Preventive HS201-PDT and Laser Exposure System. A) The large black dots represent the nipples and the stippled areas the mammary glands. Mammary glands were separated to 7 laser exposure areas, and laser was irradiated to each area starting 6 h after HS201 administration to mice (HS201: 25 nmol/mouse, DLI: 6 h, Laser dose: 30 J/cm^2 (125 mW/cm^2 x 4 min). Red circles show laser exposure areas. **B)** The black enclosure box for laser exposure is shown. Optical cable connected to the laser generator is placed inside the metal tube and the tip of the optical cable will be placed 2-3 cm above the target area of laser irradiation. The box will be closed with a metal lid before laser irradiation.

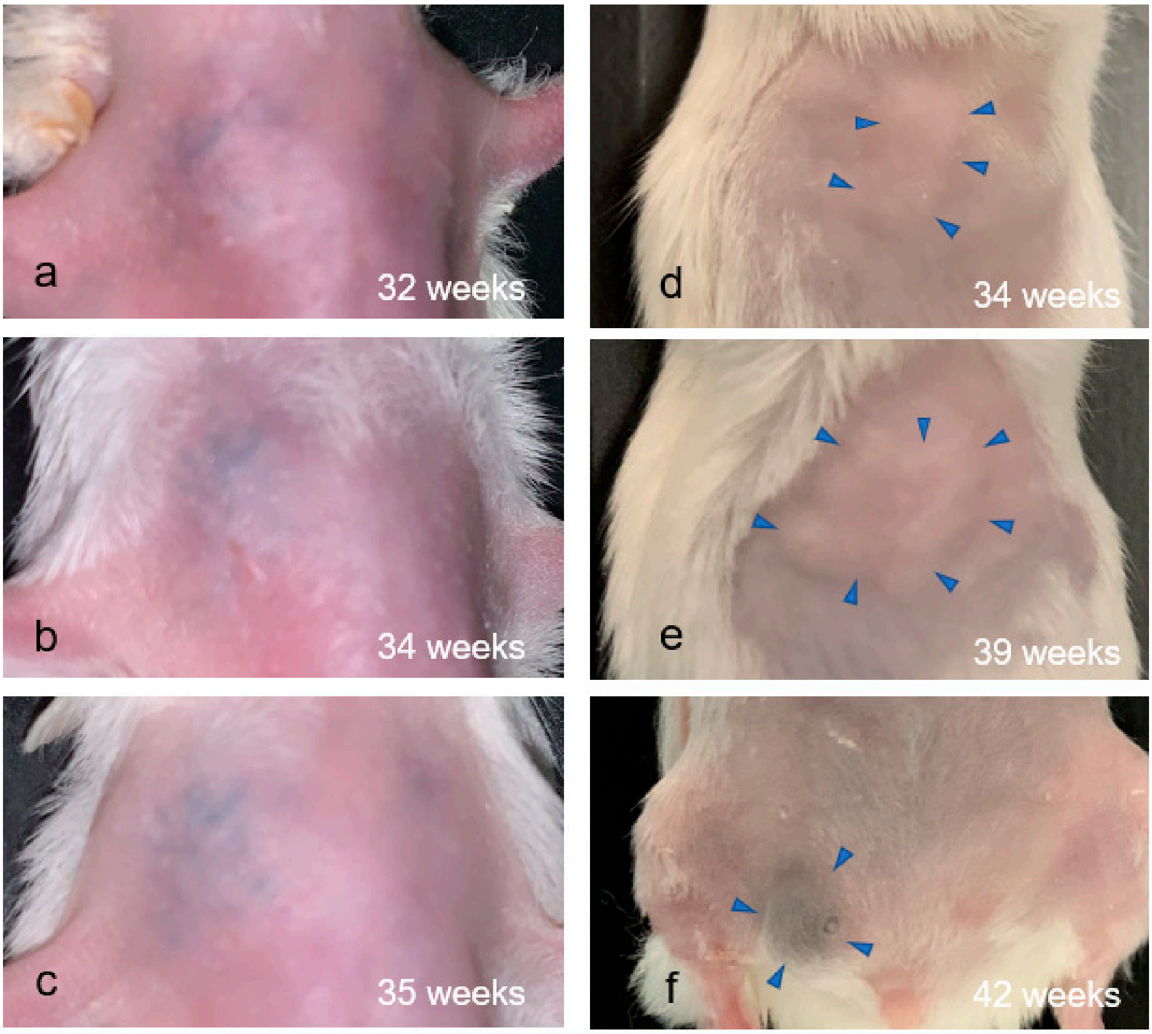

Supplementary Figure S2. Development of breast lesions in MMTV-neu mice. Representative cases of breast lesions in MMTV-neu mice are shown. (a-c) One MMTV-neu female mouse showed emergence of an intraductal proliferative lesion at the age of 32 weeks, which has spread in the right #1 mammary gland. (d) Another MMTV-neu female mouse showed soft, small and flat elevation (4 x 8 mm) in the right #3 mammary gland at the age of 34 weeks. (e) MMTV-neu female mouse at the age of 39 weeks showed soft and flat elevation (10 x 13 mm) in the right #3 mammary gland. (f) MMTV-neu female mouse at the age of 42 weeks showed a solid dark-colored tumor (7 x 8 mm) in the right #5 mammary gland.
